# Potential benefits of routine cystoscopy and vaginoscopy prior to reconstructive surgery in patients with an anorectal malformation

**DOI:** 10.1007/s00383-023-05565-0

**Published:** 2023-10-27

**Authors:** Cunera M. C. de Beaufort, Daphne T. Boom, Tara M. Mackay, Judith J. M. L. Dekker, Olga E. Arguedas Flores, Justin R. de Jong, Caroline F. Kuijper, Ramon R. Gorter

**Affiliations:** 1 Department of Pediatric Surgery, Emma Children’s Hospital Amsterdam UMC, Location University of Amsterdam, Meibergdreef 9, Amsterdam, The Netherlands; 2 Amsterdam UMC, Gastroenterology and Hepatology, Amsterdam Gastroenterology Endocrinology Metabolism, University of Amsterdam, Meibergdreef 9, Amsterdam, The Netherlands; 3 Amsterdam UMC, University of Amsterdam, Amsterdam Reproduction and Development Research Institute, Meibergdreef 9, Amsterdam, The Netherlands; 4 Department of Pediatric Urology, Emma Children’s Hospital Amsterdam UMC, Location University of Amsterdam, Meibergdreef 9, Amsterdam, The Netherlands; 5 Amsterdam UMC, Department of Surgery, Location University of Amsterdam, Meibergdreef 9, Amsterdam, The Netherlands; 6 Amsterdam UMC, Department of Gynecology, Location University of Amsterdam, Meibergdreef 9, Amsterdam, The Netherlands

**Keywords:** Anorectal malformations, Urological anomalies, Gynecological anomalies, Cystoscopy, Vaginoscopy, Screening

## Abstract

**Purpose:**

First, to assess the number of patients with anorectal malformations (ARM) in whom additional urological and/or gynecological anomalies were identified through routine screening with cysto- or vaginoscopy prior to reconstructive surgery. Second, to assess potential procedure-related complications.

**Methods:**

Retrospective mono-center cohort study, including all ARM patients born between January 2019 and December 2022. Routine screening consisted of cystoscopy for male patients, with the addition of vaginoscopy for female patients. Chi-square was used to compare the screening percentages over time.

**Results:**

In total, 38 patients were included, of whom 27 (71.1%) underwent cystoscopy ± vaginoscopy, without the occurrence of complications. Nine of 13 females (69.2%) underwent cysto- and vaginoscopy and 18 of 25 males (72.0%) underwent a cystoscopy. The percentage of patients that underwent these procedures improved over the 2 time periods (50.0% in 2019–2020 vs 90.0% in 2021–2022, p = 0.011). In 15 of 27 patients (55.6%) that underwent cystoscopy ± vaginoscopy, additional anomalies were found that were not identified through physical examination or US-kidney.

**Conclusions:**

In 56% of the patients that underwent cysto- ± vaginoscopy, additional anomalies were identified that were not with imaging studies or physical examination. This study emphasizes the potential benefit of routine cysto- and vaginoscopy in the diagnostic work-up of children with ARM.

**Level of Evidence**: III.

**Supplementary Information:**

The online version contains supplementary material available at 10.1007/s00383-023-05565-0.

## Introduction

Anorectal malformations (ARM) are rare congenital anomalies occurring in approximately 1–3 per 5000 live born children [1]. ARM can be subclassified into different types of major clinical groups and rare/regional variants, according to the Krickenbeck classification [2]. ARM can occur as an isolated anomaly, but may also be part of the VACTERL association (Vertebral, Anorectal, Cardiac, Tracheo-Esophageal, Renal, and Limb anomalies) or other underlying genetic causes or syndromes [3]. In approximately 60% of ARM patients, additional anomalies have been described, both in patients with relatively simple ARM types (e.g., recto-perineal or recto-vestibular fistula) as in more complex ARM types (e.g., recto-urethral fistula, cloacal anomalies) [4].

Additional urogenital anomalies are identified in approximately 50% of ARM patients, and can be divided into urological anomalies (i.e. urethral, bladder, ureteral, and renal anomalies) and gynecological anomalies (i.e. vaginal, cervical, and uterine anomalies) [5–7]. Furthermore, a distinction between anatomical anomalies (e.g., vaginal septum, crossed fused ectopy, and mono-kidney), and functional disorders (e.g., vesico-ureteral reflux (VUR), neurogenic bladder, and symptoms of urinary tract infection (UTI)) can be made. Besides thorough physical examination after birth, different diagnostic methods such as renal ultrasound (US-kidney), cystoscopy, vaginoscopy, voiding cysto-urethrogram (VCUG), and abdominal magnetic resonance imaging (MRI)) can be used to screen for additional urogenital anomalies in ARM patients [8–10]. Screening with cystoscopy and vaginoscopy is often performed on indication in children with complex ARM types, such as recto-urethral fistulae or cloacal anomalies, to confirm the presence and identify the level of the fistula or to measure the length of the common channel. However, cystoscopy and vaginoscopy itself might also have screening potential to identify urogenital anomalies in patients with simple ARM types (e.g., recto-perineal fistula), and in doing so, these procedures might be beneficial [11]. Therefore, in 2018, routine cysto- and/or vaginoscopy were implemented in the routine screening protocol as standard care prior to reconstructive surgery in Amsterdam UMC, for all patients with any type of ARM. For example, urethral valves might remain unidentified and potentially lead to increased bladder pressure with reflux and UTI’s as result. Nonetheless, screening protocols differ between expert centers [12].

Awareness of the potentially associated urogenital anomalies has increased over time, but discussion remains about the optimal diagnostic screening strategy and timing (e.g., in the first period of life) [6]. Comprehensive screening for additional urogenital anomalies might lead to early identification of these anomalies and subsequently improve (functional) outcomes [8, 13]. In addition, in some cases early identification of unexpected urogenital anomalies (e.g., vaginal atresia, urogenital sinus) may influence the reconstructive surgical strategy (e.g., approach and timing of the surgery) [14, 15]. Therefore, the aim of this study was to evaluated these modalities as routine screening instruments, and assess the number of ARM patients (both simple and complex types), that underwent routine screening with cystoscopy ± vaginoscopy in whom new additional urological and/or gynecological anomalies were identified. In addition, we assessed the number of patients with complications related to these procedures.

## Material and methods

### Study design and patient population

A retrospective cohort study from a database maintained since late 2018 (using Castor EDC (Electronic Data Capture) software [16]) was performed at the Emma Children’s Hospital, Amsterdam UMC. Amsterdam UMC is a tertiary referral center, accredited by the national authority as a center of expertise for ARM and a member of European Reference Network (ERN) eUROGEN. This study was designed in accordance with the Strengthening the Reporting of Observational Studies in Epidemiology (STROBE) guidelines [17]. Eligible for inclusion were all patients with any type of ARM, who were born between January 2019 and December 2022 (because routine screening with cysto- and/or vaginoscopy was implemented in late 2018), and scheduled for reconstructive surgery. Exclusion criteria were patients who died within one day after birth. Follow-up was calculated from the date of birth to the latest hospital visit or censored at date of death.

### Ethics

This study was reviewed by the medical ethical commission and was not subject to the WMO statement (ref. no. W20_230 #20.576). Regarding the primary Emma Children’s Hospital database, written information was provided to parents or legal guardians for all identified ARM patients, including a letter of objection. In case of objection, patients were removed from the database (n = 6).

### Data extraction

Data on patient characteristics, type of ARM, reconstructive surgery (e.g., anterior sagittal anorectoplasty (ASARP), posterior sagittal anorectoplasty (PSARP), other reconstructive interventions), additional anomalies (i.e. urological, gynecological, and spinal anomalies), preoperative screening (i.e. US-kidney, cystoscopy, vaginoscopy, and VCUG), peroperative findings, and complications associated with cysto- and vaginoscopy were extracted from the database by two independent researchers (CdB, DB). In case of disagreement between researchers, a third researcher (CK and/or RG) was consulted for final decision.

### Routine screening protocol for urological anomalies

**Fig. 1:**
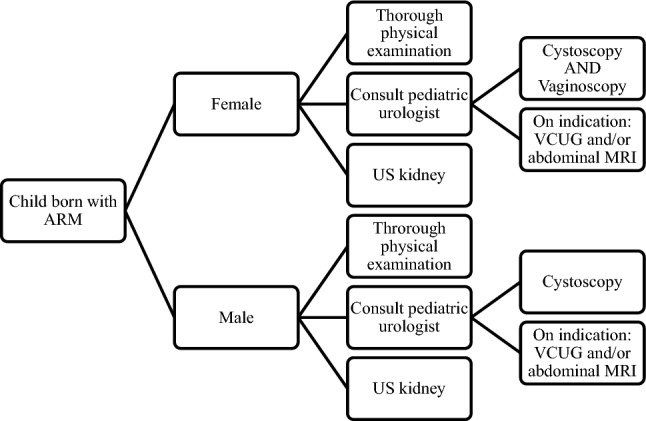
Flow diagram for Amsterdam UMC routine screening protocol to identify urogenital anomalies in patients with anorectal malformations prior to reconstructive surgery

Since 2018, routine screening to identify urogenital anomalies in Amsterdam UMC consisted of thorough physical examination to assess genital anomalies, ultrasound of the urogenital tract within 4 weeks of birth, cystoscopy (in both male and female patients), and vaginoscopy (in female patients in addition to cystoscopy) prior to the intended reconstructive surgery, proceeding immediately to reconstruction (e.g., ASARP, PSARP). Additionally, VCUG and MRI were performed on indication only. Indications for VCUG are dilated ureters and/or kidneys on US, febrile UTI, and fistula identification, and for MRI undetermined US findings or in doubt on anatomy (Fig. 1). Prophylactic antibiotics (i.e. cefazolin, metronidazole) were administered prior to the intended reconstructive surgery (e.g., ASARP, PSARP), which also covered the cystoscopy and/or vaginoscopy procedure. For cystoscopy, an 8 French scope was used, and standard assessment consisted of evaluating the presence of urethral obstruction (e.g., urethral valves), level of fistula to rectum and, aspect of the colliculus (male patients), bladderneck, ureteric ostia, trigone, and potential bladder wall trabeculation (in both male and female patients). The same 8 French scope, as used for cystoscopy, was used for vaginoscopy, and standard assessment consisted of evaluating vaginal and cervical anomalies. In case of cloacal malformations, the length of the common channel was measured. Cystoscopy and/or vaginoscopy were performed by a pediatric urologist (part of the multidisciplinary team), together with a pediatric surgeon and/or pediatric gynecologist.

### Definitions

Patients were classified according to their type of ARM using the Krickenbeck classification [2]. Urological anomalies were classified according to anatomical anomalies identified through US-kidney and/or cysto- or vaginoscopy, functional disorders identified through VCUG or by history. Gynecological anomalies were classified into anatomical anomalies, according to the European Society of Human Reproduction and Embryology (ESHRE) and the European Society for Gynecological Endoscopy (ESGE) consensus criteria [18]. Complications after cysto- and/or vaginoscopy were defined as bleeding, urethral or vaginal perforation, cervical laceration, postoperative UTI, and need for an urinary catheter ≥ 5 days. In case of uncertainties regarding classification of additional anomalies or complications, an expert panel consisting of a pediatric urologist, pediatric gynecologist, and pediatric surgeon were consulted.

### Outcomes

Primary outcome was the number of patients with ARM (both simple and complex types), that underwent routine screening with cystoscopy ± vaginoscopy, and in whom new additional urological and/or gynecological anomalies were identified prior to reconstructive surgery.

Secondary outcome was the number of ARM patients in whom complications occurred during or after routine cystoscopy and/or vaginoscopy.

### Statistical analyses

Statistical analysis was conducted using IBM SPSS Statistics for Windows, Version 28 (IBM Corp., Armonk, N.Y., USA). Descriptive statistics were used for analysis of baseline characteristics. These were reported as proportions and percentages for binary or categorical variables, and as mean with standard deviation (SD) or as median with interquartile range (IQR) for continuous variables as appropriate. To evaluate changes over time in screening, patients were categorized into 2 time periods (i.e. period 1: 2019–2020, period 2: 2021–2022). To compare screening percentages over time, because of little numbers, Fisher’s exact was used. A p value of < 0.05 was considered statistically significant. Missing or unknown data were described. No quantitative syntheses were performed due to small numbers.

## Results

### Participants

In total, 43 patients were identified in the Castor EDC database of whom 5 were excluded because no reconstructive ARM surgery was yet performed. Consequently, 38 patients were included for analysis. The cohort comprised 13 female (34.2%) and 25 male patients (65.8%) with a median age of 15.5 months (IQR 9.0–30.0) at latest follow-up. Seven different ARM types were identified, of which recto-perineal fistula occurred most often, encompassing 8 of 13 female patients (61.5%) and 13 of 25 male patients (52.0%). VACTERL association or syndromic anomalies were identified in 11 of 38 patients (28.9%), of which VACTERL-association (*n* = 3), Caudal regression syndrome (*n* = 3), and Townes − Brocks syndrome (*n* = 2) were most common. Spinal cord anomalies were identified in 9 of 38 patients (23.7%), with tethered spinal cord most often identified in 4 of 9 patients (44.4%), of whom, due to age, only 1 patient underwent urodynamic research to rule out neurogenic bladder disorder. Through physical examination, additional anomalies were identified in 2 of 13 female patients (15.4%), encompassing the presence of a vaginal tag (*n* = 1), and an enlarged clitoris and fused labia (*n* = 1), and additional anomalies were identified in 6 of 18 male patients (33.3%), encompassing hypospadia (*n* = 3), undescended testes (*n* = 3), and a bifid scrotum (*n* = 1). ASARP was the most performed reconstructive technique in 23 of 38 patients (60.5%), with median age of 3.5 months (IQR 3.0–5.25) at date of surgery. No patients underwent reconstructive surgery within the first week of life. One patient (2.6%) died during the study period due to bacterial meningitis, at 6 months of age. An overview on patient characteristics can be found in Table 1.

**Table 1:** Baseline characteristics

Sex	
Male	25 (65.8%)
Female	13 (34.2%)
Type of ARM^a^	
Recto-perineal fistula	21 (55.2%)
Recto-vestibular fistula	3 (7.9%)
Recto-urethral fistula	4 (10.5%)
Recto-vesical fistula	2 (5.3%)
Imperforate anus without fistula	3 (7.9%)
Anal stenosis	2 (5.3%)
Rare/regional variants	3 (7.9%)
Median age in months (IQR^b^)	
At latest follow-up	15.5 (9.0–30.0)
At reconstructive surgery	3.5 (3.0–5.25)
Syndrome	8 (21.1%)
VACTERL-association	3 (7.9%)
Additional spinal cord anomaly	9 (23.7%)
Mortality	1 (2.6%)

^a^*ARM* anorectal malformation. ^b^*IQR* interquartile range

### Additional imaging studies

Almost all ARM patients (35 of 38 patients (92.1%)) underwent US-kidney prior to reconstructive surgery, resulting in identification of additional anatomical urogenital anomalies in 12 of 35 patients (34.2%), with hydronephrosis (*n* = 3), bicorporeal uterus (*n* = 2), and ectopic kidney (*n* = 2) occurring most often. VCUG was performed in 8 of 38 patients (21.1%) to localize the fistula (*n* = 5), and because of dilated kidneys on US (*n* = 3), resulting in identification of VUR (bilateral grade 3–4, treated with antibiotics) in 1 of 8 patients (12.5%). A specification of patients with additional anomalies identified through US-kidney and VCUG can be found in Supplementary Material 1.

### Screening through cysto- and/or vaginoscopy

In total, 27 of 38 patients (71.7%) (9 female and 18 male) underwent routine screening with cysto- and/or vaginoscopy prior to reconstructive surgery, resulting in identification of urogenital anomalies in 15 patients (55.6%). No procedure related complications during or after cystoscopy or vaginoscopy were encountered, including no postoperative UTI’s. Over the two time periods, the percentage of patients that underwent full screening increased (*n* = 9, 50.0% vs. *n* = 18, 90.0%, *p* = 0.011). In 11 patients with recto-perineal fistula (*n* = 8), anal stenosis (*n* = 2), or imperforate anus (*n* = 1), cysto- and/or vaginoscopy were not performed without reasons available in the patient’s medical chart. In total, 23 of 35 patients (65.7%) had a urogenital anomaly (identified through either imaging studies or cysto- and/or vaginoscopy). In addition, long-term follow-up by a pediatric urologist was performed in all patients in whom any anomaly was identified through cystoscopy.

### Screening through cysto- and/or vaginoscopy in the female population

In total, 9 of 13 female patients (69.2%) underwent routine screening including cysto- and vaginoscopy prior to reconstructive surgery, resulting in identification of additional urogenital anomalies in 4 of 9 patients (44.4%). Two of 13 patients (15.4%) underwent only vaginoscopy, and 2 of 13 patients (15.4%) underwent no screening by endoscopy prior to reconstructive surgery. Through cystoscopy, additional anomalies were identified in 4 of 9 patients (44.4%), encompassing bladder wall trabeculation (*n* = 2) and a short urethra (1.2 cm) (*n* = 1). Additionally, in 1 patient (2 day old with an H-type fistula), the urethral orifice could not be identified through cystoscopy, requiring a combined cysto-vaginoscopy through the H-fistula for definitive identification of the urethral orifice. Through vaginoscopy, additional anomalies were identified in 2 of 11 patients (18.2%), encompassing vaginal atresia (*n* = 1) and a short longitudinal septum between cervices (*n* = 1). The vaginoscopy finding of vaginal atresia caused the planned reconstructive surgery (ASARP) to be postponed. An overview of female patients with additional anomalies identified in the urogenital tract through cysto- and/or vaginoscopy prior to reconstructive surgery, according to type of ARM is shown in Table 2.

**Table 2:** An overview of additional anomalies in the urogenital tract identified through cysto- and vaginoscopy according to type of ARM in females

Type of ARM^a^	Cystoscopy			Vaginoscopy		
	*n*^b^ (%)	*n*^d^ (%)	Anomalies	*n*^c^ (%)	*n*^d^ (%)	Anomalies
Recto-perineal fistula, *n* = 8	5 (62.5)	2 (40.0)	1, bladder wall trabeculation 1, short urethra	7 (87.5)	1 (14.3)	1, vaginal atresia
Recto-vestibular fistula, *n* = 3	3 (100.0)	1 (33.3)	1, bladder wall trabeculation	3 (100.0)	0 (0.0)	–
Imperforate anus without fistula, *n* = 1	0 (0.0)	0 (0.0)	–	0 (0.0)	0 (0.0)	–
Rare/regional variants, *n* = 1						
H-fistula, *n* = 1	1 (100.0)	1 (100.0)	1, urethra not identified	1 (100.0)	1 (100.0)	1, short longitudinal septum between cervices
Total, *n* = 13	9 (69.2)	4 (44.4)		11 (84.6)	2 (18.2)	

^a^*ARM* anorectal malformation. ^b^number of patients that underwent screening through cystoscopy. ^c^number of patients that underwent screening through vaginoscopy. ^d^number of patients in whom additional anomalies were identified

### Screening through cystoscopy in the male population

In total, 18 of 25 male patients (72.0%) underwent routine screening including cystoscopy prior to reconstructive surgery, identifying additional anomalies in 11 of 18 patients (61.1%), with urethral obstruction (syringocele or PUV) (*n* = 7), and bladder wall trabeculation (*n* = 3) occurring most often. All patients with PUV or syringocele underwent surgical incision by the pediatric urologist to resolve the urethral obstruction during cystoscopy, without the occurrence of complications. No planned reconstructive surgeries were postponed due to cystoscopy findings. An overview of male patients with additional anomalies in the urogenital tract identified through cystoscopy prior to reconstructive surgery according to type of ARM is shown in Table 3.

**Table 3:** An overview of additional anomalies in the urogenital tract identified through cystoscopy according to type of ARM in males

Type of ARM	Cystoscopy		
	*n* ^b^ (%)	*n* ^c^ (%)	Anomalies
Recto-perineal fistula, *n* = 13	8 (61.5)	6 (75.0)	2, bladder wall trabeculation 5, urethral obstruction
Recto-urethral fistula, *n* = 4			
Recto-prostatic fistula, *n* = 4	4 (100.0)	2 (50.0)	1, bladder wall trabeculation 1, abnormal construction colliculus, abnormal ostium
Recto-vesical fistula, *n* = 2			
Recto-vesical fistula, *n* = 1	1 (100.0)	0 (0.0)	-
Recto-bladderneck fistula, *n* = 1	1 (100.0)	1 (100.0)	1, urethral obstruction
Anal stenosis, *n* = 2	0 (0.0)	0 (0.0)	–
Imperforate anus without fistula, *n* = 2	2 (100.0)	1 (50.0)	1, syringocele
Rare/regional variants, *n* = 2			
Pouch colon, *n* = 1	1 (100.0)	1 (100.0)	1, abnormal construction colliculus
Rectal atresia, *n* = 1	1 (100.0)	0 (0.0)	
Total, *n* = 20	18 (90.0)	11 (61.1)	

^a^*ARM* anorectal malformation. ^b^number of patients that underwent screening through cystoscopy. ^c^number of patients in whom additional anomalies were identified

## Discussion

In our cohort, 27 of 38 patients (9 female and 18 male) underwent routine cysto- and/or vaginoscopy prior to reconstructive surgery, resulting in identification of additional urogenital anomalies in 44% and 61%, in the female and male population, respectively. Additional urogenital anomalies were identified in both ARM patients with both simple and more complex types. No complications during or after cysto- or vaginoscopy were encountered. In 56% of the patients that underwent cysto- and/or vaginoscopy additional anomalies were identified that were not suggested in history, seen with physical examination, or encountered on earlier imaging studies.

In this study, approximately 65% of ARM patients had a urogenital anomaly. Accurate data on the prevalence of urogenital anomalies in ARM patients is limited, but previous large cohort studies have described a prevalence of approximately 50% [8, 19–21]. Prevalence in the literature might be lower because of underreporting or –diagnosis. Most of the described anomalies in literature were identified in patients who had complaints and not in patients who underwent routine screening (i.e. with US, cysto- and/or vaginoscopy). Therefore, urogenital anomalies may be missed in these of patients. In addition, evidence is lacking regarding the number of urogenital anomalies that are identified through cysto- and vaginoscopy. In our study, the prevalence of urogenital anomalies was slightly higher compared to previous studies, possibly due to the implementation of cysto- and vaginoscopy as a routine screening procedure. This is supported by the fact that in our study more than half (56%) of the patients with a urogenital anomaly, a new urogenital anomaly was diagnosed only with cysto- and/or vaginoscopy and not through history or with imaging studies. Remarkable is that in these previous studies, without routine screening but with analysis at a later age, the prevalence of urogenital anomalies in ARM patients is comparable to our study [5, 8]. These findings underline the potential importance of screening through routine cysto- and vaginoscopy at early age instead of waiting for symptoms.

Despite implementation of routine cystoscopy ± vaginoscopy as routine screening in ARM patients since late 2018 in our center, these procedures were not performed in 7 male (28%) and 4 female (31%) patients, respectively. However, over time, the number of patients that underwent these procedures has increased greatly (to 90%). This could be explained by the fact that implementation of novel (diagnostic) strategies into clinical care pathways take some time as it first has to overcome so called obstructing factors [22]. In addition, to be successful, implementation of procedures such as cysto- and vaginoscopy should be agreed upon by the complete multidisciplinary team. Moreover, different adaptation methods and speed may be used by different physicians (i.e. early versus late adopters with different opinions on whether to perform cysto- and/or vaginoscopy in patients with more simple ARM types) which may lead to refrain from performing standard cysto- or vaginoscopy. Unfortunately, the timeframe of this study was too short to investigate this. Therefore, a follow-up study is of interest to assess implementation over time.

Embedding routine cysto- and/or vaginoscopy in standard care might be beneficial for early identification of additional urogenital anomalies in patients with ARM, both for (specific) treatment and follow-up, as well as for patient and parental counseling when anomalies are found. In this study, none of the patients suffered any hazard from the cystoscopy of vaginoscopy. In the previous studies investigating cysto- and vaginoscopy in different patients than ARM, similar results of low complication rates were reported for cysto- and vaginoscopy [23–26]. Further research should be performed to assess the financial aspects of these extra procedures prior to surgical reconstruction (i.e. longer operation time and higher costs), as well as the potential negative or positive impact that the identification of additional urogenital anomalies might have on patients and parents well-being (e.g., quality of life and quality adjusted life years) in relation to its consequences. Furthermore, in our study, in 1 patient a short urethra of 1.2 cm was identified, but measuring the urethral length with the use of cystoscopy in a small child is difficult and adequate or normal urethral lengths are not widely used and agreed upon, resulting in a remaining topic of discussion [27, 28]. Additionally, since in 1 patient the reconstructive surgery was postponed due to abnormal findings (vaginal atresia) with vaginoscopy that were not found with physical examination, it should be investigated whether surgical strategies were altered after identifying new urogenital anomalies direct prior to reconstructive surgery. Finally, future research should be performed to show if there is a possible association between types of ARM and additional urogenital anomalies.

This study should be interpreted in light of some strengths and limitations. First, to our knowledge this is the first study evaluating outcomes of standard cysto- and/or vaginoscopy prior to reconstructive surgery in ARM patients. Second, we were able to show that in half of the patients that underwent cysto- or vaginoscopy, new urogenital anomalies were identified, which were not identified through physical examination and history or with imaging studies. By incorporating routine cysto- and/or vaginoscopy prior to reconstructive surgery, this might lead to earlier diagnosis and thus earlier treatment (e.g., urethral valve or syringocele incision, septal cleaving for vaginal septum). However, although cysto- and vaginoscopy were performed by a specialized multidisciplinary team with a key role for the pediatric urologist, the identified urogenital anomalies might be subject to inter observer variability. Furthermore, due to the retrospective nature of this study, information and selection bias are likely present. To reduce this to a minimum, data was extracted by the primary researcher (CB) with a data check by another coauthor (TM) and all patients were consecutively included between 2019 and 2022. Because ARM is extremely rare, only a small number of patients could be included and extensive statistical analyses were not possible. Even though numbers are small, it is important to report these data, to open a discussion on whether or not routine cysto- and/or vaginoscopy is indicated and of importance as standard care in patients with ARM.

In conclusion, 71% of our ARM patients underwent routine cysto- and vaginoscopy in female patients and cystoscopy in male patients prior to reconstructive surgery, without occurrence of complications. In more than half of the patients that underwent these procedures, new urogenital anomalies were identified through cysto- or vaginoscopy that were not found with history, physical examination or imaging studies. This emphasizes the potential benefit and importance of routine cystoscopy and/or vaginoscopy in the diagnostic work-up prior to reconstructive surgery in patients with ARM.

### Supplementary Information

Below is the link to the electronic supplementary material.

Supplementary file1 (DOCX 16 KB)

## Data Availability

Requests for data sharing will be considered upon written request to the corresponding author.
