# Supplementary material for: Potential benefits of routine cystoscopy and vaginoscopy prior to reconstructive surgery in patients with an anorectal malformation
Source: Pediatr Surg Int. 2023 Oct 27;39(1):284. doi: 10.1007/s00383-023-05565-0 (PMC10611826; doi:10.1007/s00383-023-05565-0)
Supplement: Supplementary file 1 — Supplementary file1 (DOCX 16 KB) [file 383_2023_5565_MOESM1_ESM.docx]

Supplementary Table 1a. An overview of number of female patients with anatomical and functional additional anomalies in the urogenital tract identified through US-kidney and VCUG according to type of ARM.

| **Type of ARM^a^** | **US-kidney^b^** | | | **VCUG^c^** | |
| --- | --- | --- | --- | --- | --- |
|  | n^d^ (%) | n^e^ (%) | Anomaly | n^f^ (%) | n^e^ (%) |
| Recto-perineal fistula, n=8 | 6 (75.0) | 1 (16.7) | Mono-kidney | 0 (0.0) | 0 (0.0) |
| Recto-vestibular fistula, n=3 | 3 (100.0) | 1 (33.3)  1 (33.3) | Adrenal gland anomaly  Bicorporeal uterus | 1 (33.3) | 0 (0.0) |
| Imperforate anus without fistula, n=1 | 0 (0.0) | 0 (0.0) |  | 0 (0.0) | 0 (0.0) |
| Rare/regional variants, n=1 |  |  |  |  |  |
| H-fistula, n=1 | 1 (100.0) | 1 (100.0) | Bicorporeal uterus, hydrocolpos | 1 (100.0) | 0 (0.0) |
| **Total, n=13** | 10 (76.9) | 4 (40.0) |  | 2 (15.4) | 0 (0.0) |

^a^ ARM= anorectal malformation.

^b^ US-kidney= renal ultrasound.

^c^ VCUG= voiding cysto-urethrogram.

^d^ number of patients that underwent screening through US-kidney.

^e^ number of patients in whom additional anomalies were identified.

^f^ number of patients that underwent screening through VCUG.

Supplementary Table 1b. An overview of number of male patients with anatomical and functional additional anomalies in the urogenital tract identified through US-kidney and VCUG according to type of ARM.

| **Type of ARM^a^** | **US-kidney^b^** | | | **VCUG^c^** | |
| --- | --- | --- | --- | --- | --- |
|  | n^d^ (%) | n^e^ (%) | Anomaly | n^f^ (%) | n^e^ (%), anomaly |
| Recto-perineal fistula, n=13 | 13 (100.0) | 1 (7.8)  1 (7.8)  1 (7.8)  1 (7.8) | Bilateral cortical microcysts  Bilateral nefrocalcinosis  Hydronephrosis  Mono-kidney | 2 (15.4) | 0 (0.0) |
| Recto-urethral fistula, n=4 |  |  |  |  |  |
| Recto-prostatic fistula, n=4 | 4 (100.0) | 2 (50.0) | Ectopic kidney  Hydronephrosis | 1 (25.0) | 0 (0.0) |
| Recto-vesical fistula, n=2 |  |  |  |  |  |
| Recto-vesical fistula, n=1 | 1 (100.0) | 1 (100.0) | Hydronephrosis, testis intra-abdominal | 0 (0.0) | 0 (0.0) |
| Recto-bladderneck fistula, n=1 | 1 (100.0) | 0 (0.0) |  | 0 (0.0) | 0 (0.0) |
| Anal stenosis, n=2 | 2 (100.0) | 0 (0.0) | - | 0 (0.0) | 0 (0.0) |
| Imperforate anus without fistula, n=2 | 2 (66.7) | 0 (0.0) | - | 1 (50.0) | 1 (50.0) VUR grade 3-4^g^ |
| Rare/regional variants, n=2 |  |  |  |  |  |
| Pouch colon, n=1 | 1 (100.0) | 0 (0.0) | - | 1 (100.0) | 0 (0.0) |
| Rectal atresia, n=1 | 1 (100.0) | 1 (100.0) | Ectopic kidney | 1 (100.0) | 0 (0.0) |
| **Total, n=25** | 25 (100.0) | 8 (32.0) |  | 6 (24.0) | 1 (16.7) |

^a^ ARM= anorectal malformation.

^b^ US-kidney= renal ultrasound.

^c^ VCUG= voiding cysto-urethrogram.

^d^ number of patients that underwent screening through US-kidney.

^e^ number of patients in whom additional anomalies were identified.

^f^ number of patients that underwent screening through VCUG.

^g^ VUR= vesico-urethral reflux.
